# Cellular profile of hematopoietic stem cells mobilized by exercise versus G-CSF in healthy related donors

**DOI:** 10.1007/s00421-026-06224-7

**Published:** 2026-04-10

**Authors:** Elias Siebold, Laura Infanti, Hans-Jürgen Gruber, Gregor T. Stehle, Debbie Maurer, Egbert T. Ritter, Michael Medinger, Francesca Matteazzi, Arno Schmidt-Trucksäss, Andreas Holbro, Julia M. Kröpfl

**Affiliations:** 1 Division of Sport and Exercise Medicine, Department of Sport, Exercise and Health, University of Basel, Grosse Allee 6, Basel, 4052 Switzerland; 2 Blood Transfusion Center North-Western Switzerland, Swiss Red Cross, Hebelstrasse 10, Basel, 4056 Switzerland; 3 Clinic of Hematology, University Hospital Basel, Petersgraben 4, Basel, 4031 Switzerland; 4 Clinical Institute of Medical and Chemical Laboratory Diagnostics, Medical University of Graz, Auenbruggerplatz 15, Graz, 8036 Austria; 5 Clinic of Internal Medicine III: Oncology, Hematology and Palliative Medicine, Diakoniestrasse 10, Schwäbisch Hall, Germany

**Keywords:** Stem cell transplantation, Apheresis, Granulocyte colony-stimulating factor (G-CSF), Exercise-induced hematopoietic stem cell mobilization

## Abstract

**Purpose:**

Hematopoietic stem cell transplantation (HSCT) depends on effective HSC mobilization with granulocyte colony-stimulating factor (G-CSF), though some allogeneic donors experience severe side effects. This study compares G-CSF and acute maximal exercise for HSC collection and cellular composition in 10 healthy family donors, examining links to donor fitness and recipient outcomes.

**Methods:**

Ten donors (3 females; 40.8 (18.3) years) and their recipients (*n* = 8; 3 females; 47.5 (18.5) years) were enrolled. Donors completed cardiopulmonary exercise testing with blood samples taken pre- and post-exercise and on apheresis day for flow cytometric analysis. Recipients were followed up on day ~ 30 post-transplant.

**Results:**

Exercise significantly increased circulating total CD34+ cells (1.76-fold (0.51), *p* = 0.002) and select progenitor subsets (CD34+CD45dim, 1.74-fold (0.56), *p* = 0.006; and CD34+CD38+CD133+, 1.60-fold (0.46), *p* = 0.013), while other subsets showed no significant change (*p* > 0.05). Compared with post-exercise, apheresis resulted in markedly higher concentrations of total CD34+ cells (20.7-fold (9.7), *p* < 0.001) and all measured subsets. The largest enrichments were observed in CD34+ CD38−CD133+ (52.7-fold (69.3) *p* = 0.005) and CD34+CD38+CD133+ (38.2-fold (17.0), *p* < 0.001) populations. HSC collection at apheresis contained a 186.1-fold (72.3) elevation of immature granulocytes (*p* < 0.001) compared to the acute exercise (1.5-fold (0.3)). The proportions of apoptotic CD34+ cells and their subpopulations were similar post-exercise to apheresis (*p* > 0.05). Donor CD34+CD38- cell number was significantly negatively associated with recipient engrafting CD34+CD45dimCD90+ cells/µl (*r*=-0.70, *p* = 0.05, *n* = 8).

**Conclusion:**

Exercise might complement pharmacological mobilization by enhancing key HSC subgroups, improving collection quality, and supporting transplantation success; future research should evaluate combined approaches and the role of CRF in donor suitability and recipient outcomes.

**Supplementary Information:**

The online version contains supplementary material available at https://doi.org/10.1007/s00421-026-06224-7.

## Introduction

Hematopoietic stem cell transplantation (HSCT) is a potentially curative therapy and standard of care for selected patients with hematologic malignancies, including high-risk acute myeloid leukemia, as well as certain non-malignant hematologic and immune disorders, with its use steadily increasing (Khwaja et al. 2016). HSCT involves the infusion of bone marrow or peripheral hematopoietic stem cells (HSC, CD34+ cells) from a donor following cytotoxic and immunosuppressive therapy. To enhance HSC mobilization in the peripheral blood, granulocyte colony-stimulating factor (G-CSF) is commonly administered for 4–5 days before HSC collection at approximately 10 µg/kg/day (Salhotra et al. 2023). However, some allogeneic donors fail to reach the minimum CD34 + cell threshold of 2 × 10⁶ per kg of recipient body weight, despite G-CSF administration (Bazinet and Popradi 2019). Plerixafor, a newer mobilizing agent, has demonstrated efficacy in improving HSC yields, particularly when combined with G-CSF (Teipel et al. 2018), yet its high cost restricts widespread use to poor mobilizers and is mainly used for related donors (Romon et al. 2022; Miyazaki and Suzuki 2018). Both G-CSF and plerixafor are generally safe, with serious adverse effects like splenic rupture being rare, while more common side effects include bone pain, nausea, headache, and fatigue (Salhotra et al. 2023; Romon et al. 2022).

Several factors are associated with reduced CD34+ cell mobilization, including female sex, low body mass index (BMI), advanced age, and smoking, though poor mobilization can also occur without these risk factors (Teipel et al. 2015; de Kruijf et al. 2020; Kongtim and Ciurea 2019). The CD34+ cell count per kg of recipient body weight determines graft sufficiency, but graft composition also plays a crucial role in engraftment, though its impact remains under-researched (Salhotra et al. 2023). Additionally, the CD34+ population is heterogeneous, with subsets differing in differentiation potential (de Kruijf et al. 2020; Calloni et al. 2013). Key markers - such as CD110, CD38, and CD133 - further define HSC subsets being capable of multilineage differentiation (Calloni et al. 2013; Ninos et al. 2006). CD34+ subsets like CD34+CD110+, CD34+CD38−, and CD34+CD133+ have been associated with shorter engraftment times and improved clinical outcomes (Henon et al. 2001; Camacho Villa et al. 2012; Sartor et al. 2007).

Physiological stress, such as exhaustive exercise, transiently increases circulating CD34+ cells, with effects lasting up to 48 h depending on the subpopulation (Schmid et al. 2021). This rapid mobilization suggests that HSC originate from vascular marginal pools rather than directly from bone marrow, as bone marrow-derived HSC take days to appear in circulation post-G-CSF therapy. Mobilization is driven by β2-adrenoreceptor signaling and hemodynamic forces from increased cardiac output and blood flow (Agha et al. 2018; Kroepfl et al. 2012; Niemiro et al. 2017; Simpson et al. 2021). Given its non-invasive nature with no severe side effects, acute exercise of different intensities is being explored as an adjuvant for HSC mobilization (Cox et al. 2025), particularly during G-CSF treatment, to enhance HSC yields before or during apheresis (Simpson et al. 2017; Emmons et al. 2016; Gustafson et al. 2021; Wadley et al. 2025; McDougal et al. 2025). However, a direct comparison of both mobilization regimens in the same donor has yet to be elucidated.

A higher cardiorespiratory fitness (CRF) should also prime HSC in bone-marrow and make them less “susceptible” to physiological stressors (De Lisio and Parise 2013). This is called the “hormetic effect” of exercise training, which has previously been shown to enhance cellular protection (De Lisio et al. 2011) and to reduce inflammatory cell production via instruction of HSC (Frodermann et al. 2019). In addition, higher CRF is typically associated with lower BMI and a reduced pro-inflammatory cytokine profile. Since greater baseline inflammatory signaling has been linked to enhanced mobilization efficacy (Wang et al. 2022), a lower inflammatory milieu may theoretically attenuate responsiveness to mobilizing stimuli. These adaptations could extend beyond endogenous physiological stressors to exogenous agents such as G-CSF. Consequently, it is plausible to hypothesize that donors with higher CRF exhibit reduced sensitivity to G-CSF–induced mobilization and may therefore represent comparatively poorer mobilizers.

Moreover, donor CRF may influence specific CD34+ subpopulations critical for favorable transplantation outcomes, a hypothesis that remains to be tested. While bone marrow from exercised mice did not enhance HSC homing, engraftment, or hematopoietic reconstitution, the number of spleen colonies seven days post-transplant was 48% higher than in recipients of marrow from sedentary mice (De Lisio and Parise 2012), suggesting improved splenic function and potentially faster neutrophil and platelet recovery (Akpek et al. 2013). This effect may have been overlooked due to the absence of differential counts and CD34+ analyses, and validation in humans is still required. A deeper understanding of early hematopoietic regeneration in recipients remains an important knowledge gap (Bájecný et al. 2021).

The primary aim of this study is to compare the mobilization of HSC using G-CSF and an exhaustive bout of maximal cycling exercise. Specifically, the study focuses on cell proportions and concentration estimates and their apoptotic subsets generated by the two mobilization strategies. Secondary aims are to evaluate if a higher CRF plays any role for (1) mobilization efficacy in the donor and (2) engrafting cells and/or quality of life (QoL) outcome parameters in the related recipient.

We hypothesize that acute exercise produces less apoptotic cell proportions and that a higher CRF is associated with less mobilized HSC by either mobilization regimen. Furthermore, higher donor CRF will be positively correlated with recipient engrafting CD34+CD45dimCD90+ cells and QoL outcomes.

## Methods

### Ethics approval

The study was approved by the local ethics committee (EKNZ ID: 2022-01624). All participants received detailed information about the study procedures and provided written informed consent prior to participation.

### Recruitment and study procedure

Participants were recruited through the Blood Transfusion Center North-Western Switzerland. Only healthy related donors of peripheral HSC collection and their respective recipients were considered for the study. Eligible participants were informed about the nature and objectives of the study, and those donors who agreed to participate were invited to the Department of Sports, Exercise and Health (DSBG) of the University of Basel prior to their HSC donation. Each donor underwent a standardized assessment procedure, which included three blood withdrawals at different time points, a body composition analysis, CRF assessment via a cardiopulmonary exercise test (CPET), and physical activity assessment via accelerometer and questionnaire analyses. Recruitment occurred at the donor medical check scheduled within 30 days of the donation. The DSBG visit was scheduled between the donor check and day − 12. At the DSBG visit, participants also underwent a brief medical examination. Participants were instructed to arrive well rested, having obtained at least 7 h of sleep on each of the two nights preceding their visit. They were asked to refrain from vigorous physical activity for 48 h and from any general physical activity for 24 h prior to testing. In addition, participants were required to abstain from alcohol consumption for 48 h before their visit and from caffeine consumption on the day of testing prior to the assessments. Body composition was assessed using the InBody 720 analyzer (InBody Co. Ltd., Seoul, South Korea), along with measurements of height using a stadiometer. A lung function test was conducted to confirm participants’ ability to complete the CPET. The day of HSC collection was designated as day 0, with the first dose of G-CSF administered on day − 4. Blood samples were collected before (PRE) and directly after the CPET (POST), with an additional blood withdrawal at day 0 at the Blood Transfusion Center directly before the apheresis procedure (APHERESIS). Blood sampling was standardized as far as feasible. In the PRE condition, venous blood was collected between 09:05 and 11:15 under resting conditions in a non-fasted state. In the POST condition, samples were obtained between 09:55 and 12:45, approximately 4.08 to 7.55 min after cessation of exercise. In the APHERESIS condition, donors received no specific instructions for the day(s) preceding HSC collection. The apheresis procedure was initiated between 08:30 and 09:00. Blood samples were collected immediately after venipuncture at the start of the procedure within this time window. After HSCT an additional blood collection in the respective recipients was performed at day *~* 30 post-transplant. At the same day, recipient QoL was investigated by the MOS 36 item short-form health survey (SF-36) (McHorney et al. 1994).

**Fig. 1:**
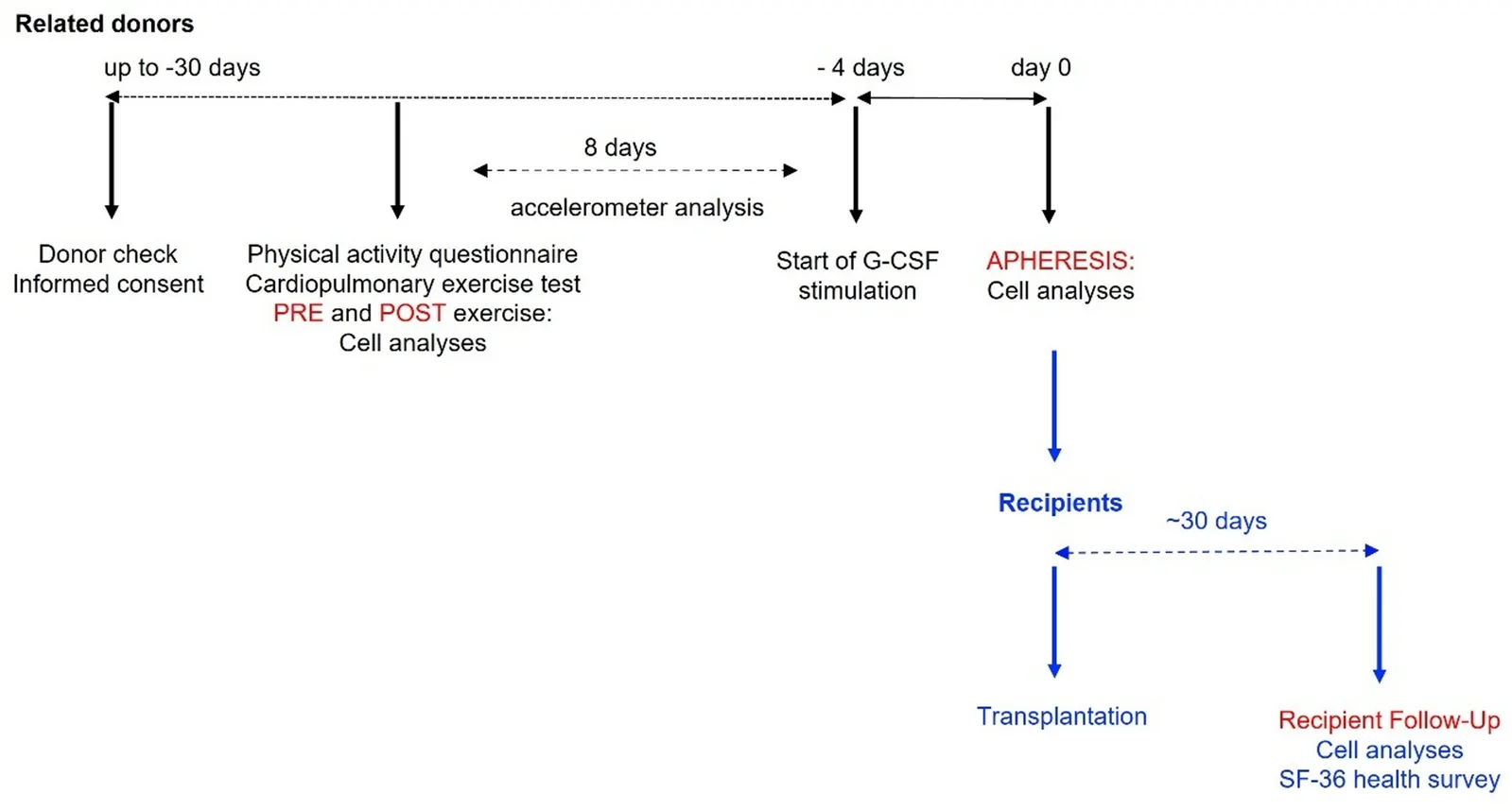
Study design of the StemCellFit study

Please find an overview of the study design in Fig. 1.

### Blood preparation and analysis

**Fig. 2:**
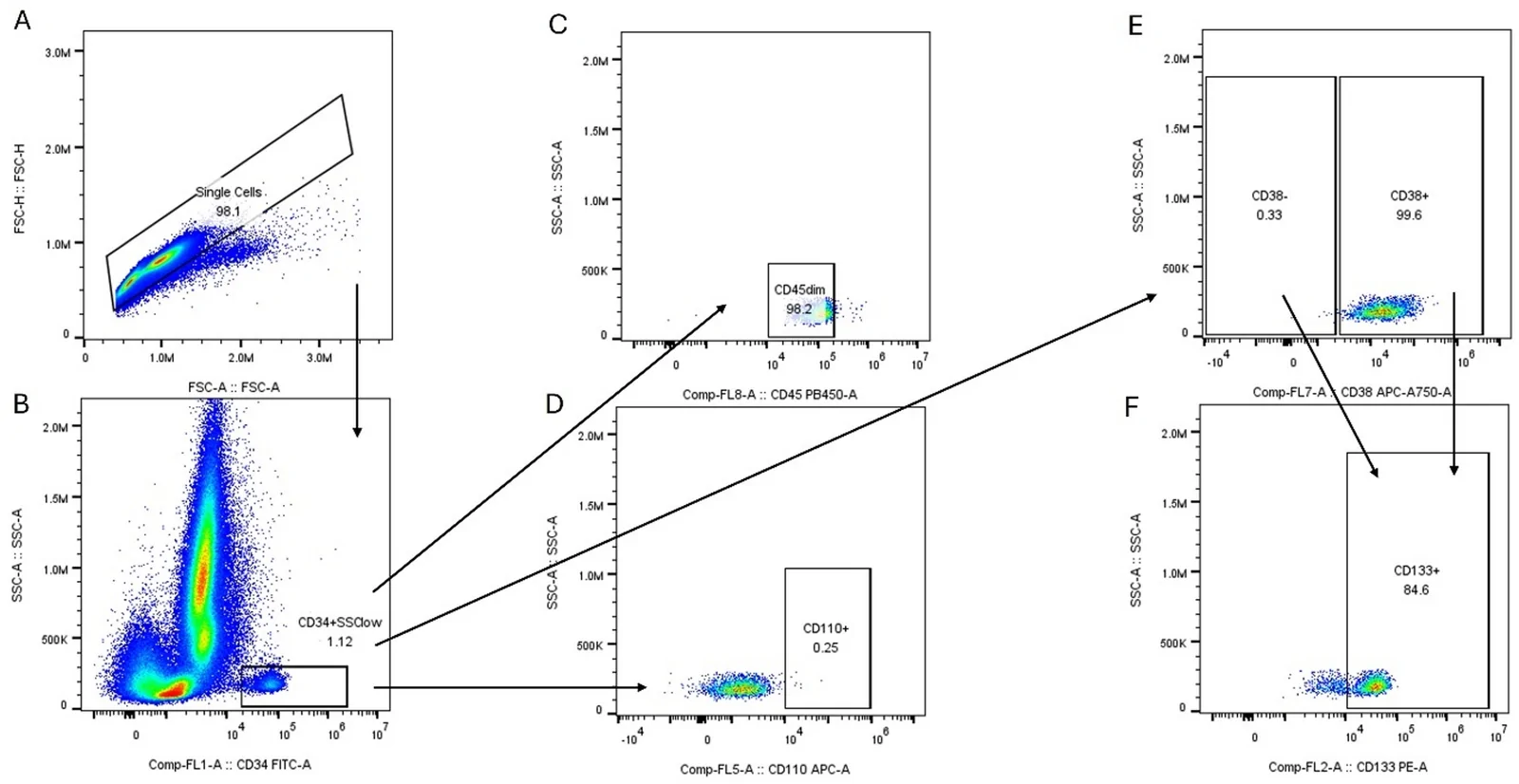
Donor flow cytometry exemplary staining. After doublets exclusion (**A**), total CD34+ cells are defined as side scatter low (SSClow, **B**). CD34+ subgroups are subsequently extracted by CD45dim (**C**) and CD110 expression (**D**) or CD38 (**E**) and CD133 expression (**F**). In case of total CD34+SSClow cells, numbers represent the percentage of MNC; for all respective subgroups, numbers express the percentage of total CD34+ cells

Each blood collection involved two 7.5 mL EDTA tubes (Sarstedt, Sevelen, Switzerland). One was used for complete blood counts (CBC); the other for mononuclear cell (MNC) staining. CBCs were performed on the Sysmex XN-350 (Sysmex Suisse AG, Horgen, Switzerland), providing counts for white blood cells (WBC), red blood cells (RBC), platelets (PLT), neutrophils (NEUT), lymphocytes (LYMPH), monocytes (MONO), eosinophils (EO), basophils (BASO), and immature granulocytes (IG). The remaining blood in the second tube was centrifuged (1300 rpm, 20 °C, 10 min), and the plasma was stored at -80 °C until further analysis. Granulocyte colony-stimulating factor (G-CSF) was analyzed in plasma using a standard ELISA procedure (Thermofisher Scientific, Allschwil, Switzerland). HSC detection and enumeration was done as previously described (Kropfl et al. 2019; Duda et al. 2007). After MNC isolation by density gradient centrifugation, donor cells were stained with antibodies against CD34-FITC, CD38-APC-Cy7, CD45-BV421, CD110-Alexa647, and CD133-PE (BD Biosciences, Allschwil, Switzerland). Recipient cells were stained with CD34-FITC, CD45-BV421, CD90-Alexa647 (BD Biosciences, Allschwil, Switzerland). For apoptosis analysis, cells were incubated with Live/Dead Fix Aqua (Life Technologies, Zug, Switzerland), Annexin V-PerCP-Cy5.5, and Annexin binding buffer (BD Biosciences, Allschwil, Switzerland) at room temperature. After fixation, flow cytometry was performed on a Cytoflex device (Beckmann Coulter, Nyon, Switzerland). Cell subsets were categorized as viable, early/late apoptotic, or dead. Values of viable and apoptotic CD34⁺ cells and their subsets were calculated and expressed as percentages of the total CD34+ population. Relative proportions of total CD34+ cells are reported as percentages of MNC, whereas CD34+ subpopulations are expressed as a percentage of total CD34+ cells. Absolute cell concentrations, expressed as cells/µL, were estimated using a dual-platform approach based on whole blood cell counts obtained with the hemolyzer (Sutherland et al. 1996). Fluorescent-minus-one controls, and compensation beads (BD Biosciences, Allschwil, Switzerland) ensured gating accuracy and spill-over correction, respectively. Flow cytometry datasets were processed and analyzed using FlowJo software (FlowJo LLC, Oregon, USA). An overview of the gating strategy is presented in Fig. 2.

### Donor cardiorespiratory fitness and physical activity assessment

Cardiorespiratory fitness was assessed via CPET, performed as a ramp protocol until exhaustion using an ergometer (Sport Excalibur, Lode Medical Technology, Groningen, The Netherlands), and the PowerCube spirometer (Ganshorn Medizin Electronic, Niederlauer, Germany). The ramp protocol was selected based on donors’ fitness status (protocol II: warm-up 10 W, then increase of 10 W/min; protocol III: warm-up 20 W, then increase of 15 W/min; protocol IV: warm-up 50 W, then increase of 20 W/min; protocol V: warm-up 50 W, then increase of 30 W/min). The same day, donors were given a triaxial accelerometer (GENEActiv; Activinsights Ltd., Kimbolton, UK) to wear on their nondominant wrist for 24 h per day over 8 days. Data were sampled at 50 Hz. Days with wear time > 14 h were taken as valid. Donors needed to have at least four valid weekdays (Monday to Friday) and one weekend day for analysis (Schwendinger et al. 2024). Physical activity was also investigated via questionnaire (GPAQ) (Keating et al. 2019).

### Statistics

Data are given as mean (standard deviation) or median (in radial plots). A-priori power analysis (comparison between (Teipel et al. 2018) and (Baker et al. 2017) revealed a total sample size of *n* = 8 (paired t-test, effect sizes = 1.39; alpha = 0.05 and 1-beta > 0.8) to find a significant difference between G-CSF-induced and exercise-induced CD34+ cell number. Paired t-tests or Wilkoxon-tests were used to compare total, viable and apoptotic CD34+ cell counts and subsets as well as CBC parameters across conditions depending on distributions of the paired differences. Fold changes between conditions were computed to assess relative mobilization effects. Results were corrected for multiple testing by the Bonferroni method. Associations were investigated using Pearson, Spearman, or repeated measures correlation (coefficient r_rm_) analysis depending on variable distributions. Unlike simple correlation, repeated measures correlation analysis does not violate the assumption of independence of observations and generally offers greater statistical power in repeated measures designs. The slopes displayed identify the best fit for all intraindividual associations (Bakdash and Marusich 2017). Calculations were performed using R version 3.3.0 and the “rmcorr” package. All other analyses and graphical presentations were executed using Excel, Python (Version 3.11.7) and IBM SPSS Statistics (Version 30.0.0.0). Statistical significance was defined as a two-sided p-value of ≤ 0.05.

## Results

### Descriptive statistics of donors and their respective recipients

All donors (*n* = 10, 3 females) completed all three blood withdrawals at pre-, and post-exercise as well as at apheresis, with successful measurements of CBC and HSC. Donors were on average 40.9 (18.1) years old with a BMI of 26.0 (3.9) kg/m^2^. Average skeletal muscle mass was 32.5 (7.6) kg, fat mass was 22.3 (10.8) kg, and body fat percentage was 27.6 (11.4)%. The mean waist-to-hip ratio (WHR) was 0.954 (0.097). Only *n* = 8 transplant recipients could be included in the study (3 females, age 47.5 (18.5); BMI 25.4 (4.0)), the respective recipient of SCF01 was underage and the one of SCF06 unfortunately died. Please find individual donor and recipient characteristics as well as the indications for allo-HSCT in Supplement Table S1.

### G-CSF analysis

The acute exercise did not significantly change G-CSF from pre- (336.7 (264.2) pg/ml) to post-exercise (316.6 (235.3) pg/ml, *p* = 0.360), while it increased at apheresis compared to post-exercise (6471.1 (1838.6) pg/ml, *p* < 0.001).

### Whole blood count

**Fig. 3:**
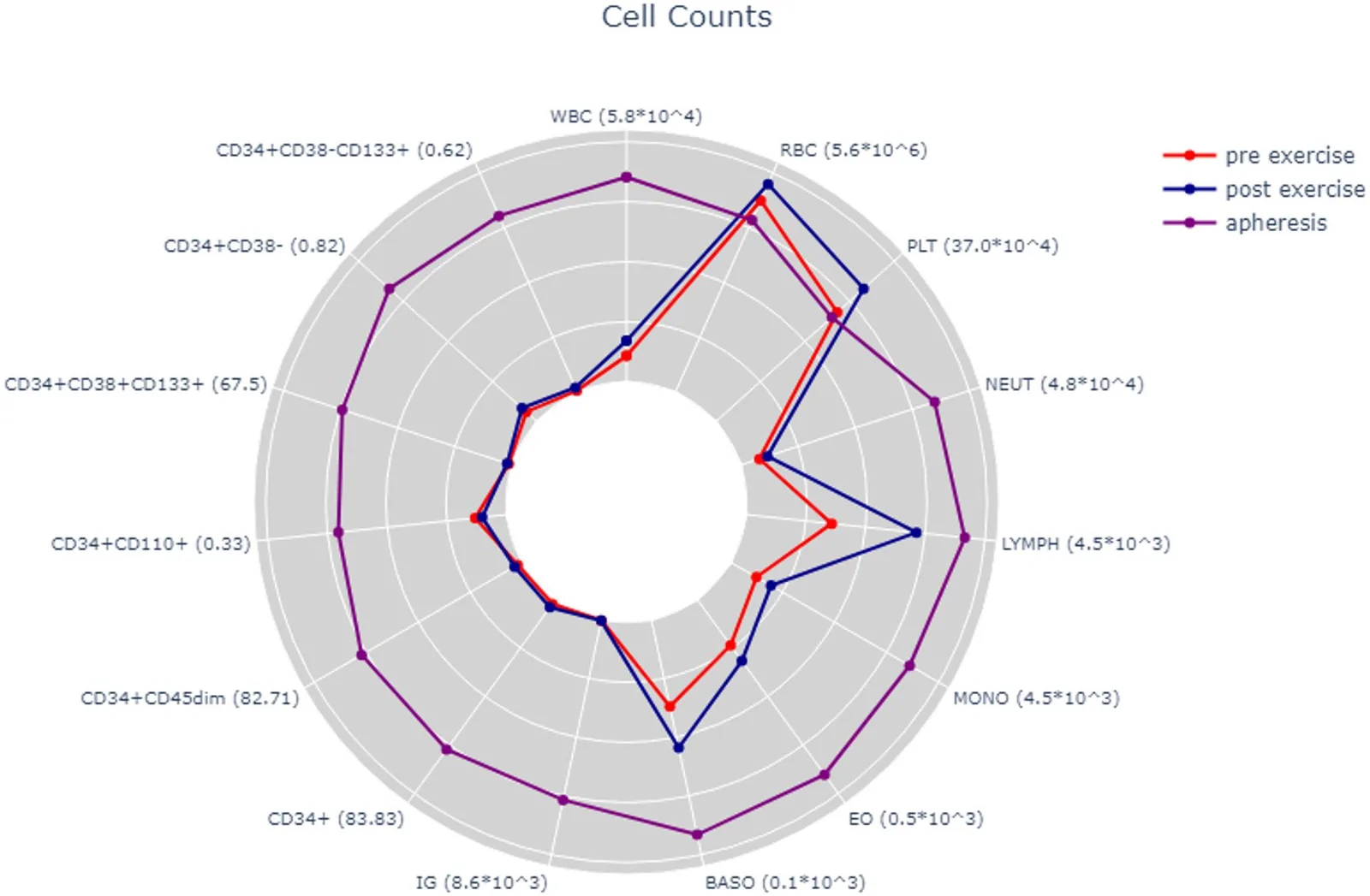
Radial plot of cell concentration estimates (cells/µL) induced by acute exercise vs. apheresis. Each cell group is scaled according to the highest third quartile value (pre-exercise, post-exercise, apheresis), which is indicated in parentheses. The figure presents median values for each cell group across all conditions. Subsets of CD34+ cells are specified as CD34+CD45dim, CD34+CD110+, CD34+CD38+CD133+, CD34+CD38-, and CD34+CD38-CD133+. *WBC*, white blood cells; *RBC*, red blood cells; *PLT*, platelets; *NEUT*, neutrophils; *LYMPH*, lymphocytes; *MONO*, monocytes; *EO*, eosinophils; *BASO*, basophils; *IG*, immature granulocytes;

Comparing pre- and post-exercise conditions, WBC (1.6-fold (0.2), *p* < 0.001), LYMPH (2.1-fold (0.4), *p* < 0.001), MONO (1.6-fold (0.3), *p* = 0.005), NEUT (1.5-fold (0.1), *p* < 0.001), EO (1.4-fold (0.3), *p* < 0.001), BASO (1.6-fold (0.3), *p* < 0.001), IG (1.5-fold (0.3), *p* = 0.004), as well as RBC (1.1-fold (0.04), *p* = 0.005), and PLT (1.2-fold (0.2), *p* = 0.002) significantly increased after exercise cessation (Fig. 3).

At HSC collection, there was another rise in cell concentrations from the post-exercise condition for WBC (5.0-fold (0.7), *p* < 0.001), NEUT (7.7-fold (1.8), *p* < 0.001), MONO (4.5-fold (1.3), *p* < 0.001), EO (3.6-fold (2.4), *p* < 0.001), BASO (2.0-fold (1.1), *p* = 0.001), and LYMPH (1.3-fold (0.3), *p* = 0.022). RBC and PLT were reduced at apheresis compared to post-exercise (0.8-fold (0.1), *p* = 0.005; 0.9-fold (0.5), *p* = 0.010, respectively). Notably, there was a 186.1-fold (72.3) elevation in IG from post-exercise to apheresis (*p* < 0.001).

### Total CD34+ cells and subsets as concentration estimates

Total CD34+ (1.76-fold (0.51), *p* = 0.002), as well as CD34+CD45dim (1.74-fold (0.56), *p* = 0.006), and CD34+CD38+CD133+ (1.60-fold (0.46), *p* = 0.013) cell subsets significantly increased in concentration from pre- to post-exercise (Fig. 3), while CD34+CD38- (2.50-fold (2.88), *p* = 0.088), CD34+CD38-CD133+ (2.12-fold (2.02), *p* = 0.075), and CD34+CD110+ (0.84-fold (0.50), *p* = 0.093) did not change.

Total CD34+ cells and their subsets exhibited an increase in concentration at apheresis compared to post-exercise conditions (Fig. 3). Specifically, total CD34+ concentration estimates increased from post-exercise (3.46 (2.03) cells/µL) to apheresis (64.80 (30.38) cells/µL) 20.7-fold (9.7) (*p* < 0.001). Similar or even higher increases were observed for CD34+CD45dim (23.8-fold (11.9), *p* < 0.001), CD34+CD38+CD133+ (38.2-fold (17.0), *p* < 0.001), and CD34+CD38-CD133+ (52.7-fold (69.3) *p* = 0.005). CD34+CD38- and CD34+CD110+ cell subsets showed smaller elevations (9.3-fold (8.1), *p* = 0.005; 8.0-fold (5.4), *p* < 0.001; respectively).

### Viable and apoptotic subsets as percent of total CD34+ cells

**Fig. 4:**
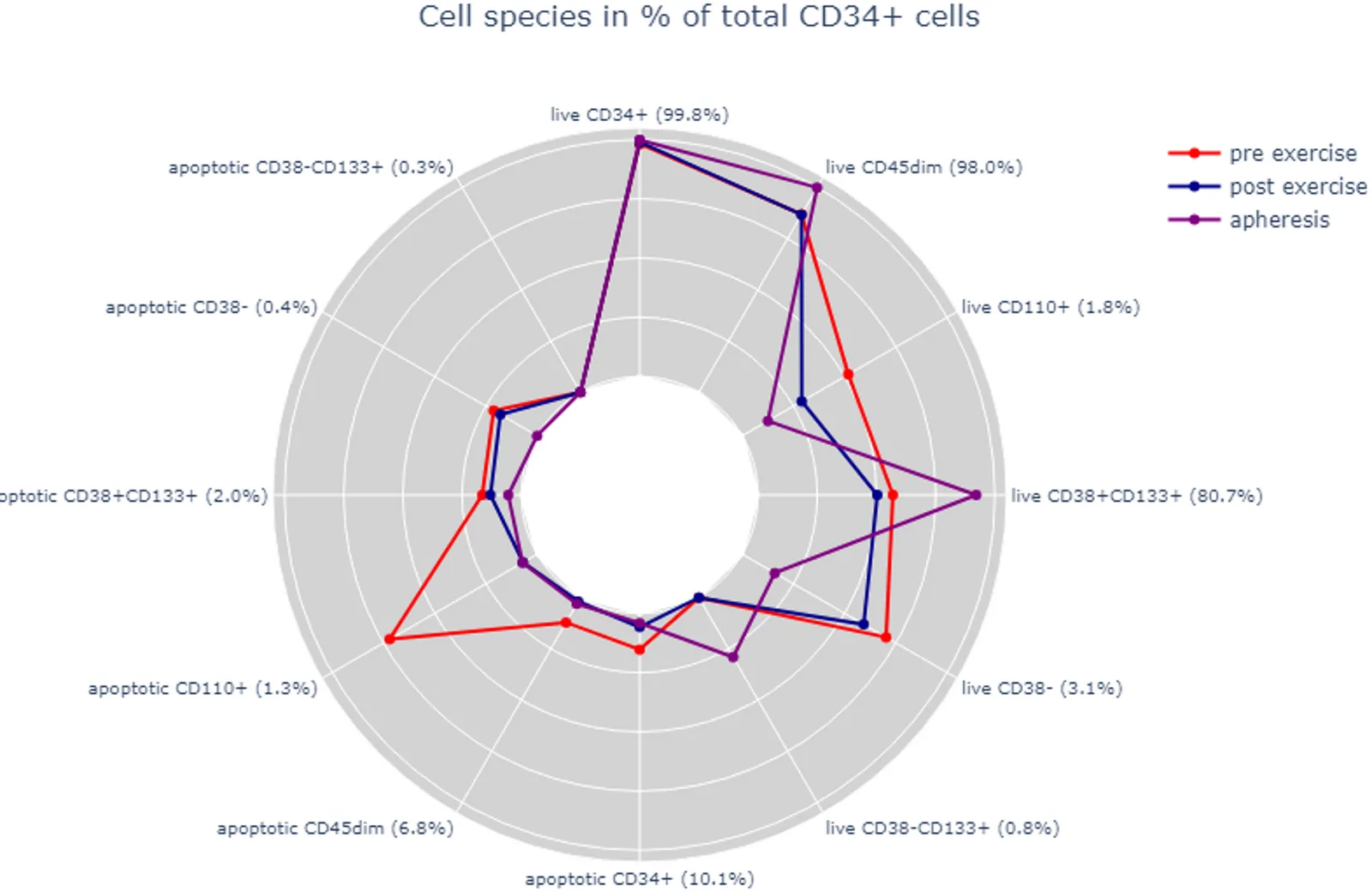
Radial plot of viable and apoptotic cell subsets in % of total CD34+ cells induced by acute exercise vs. apheresis. Percentages of each CD34+ cell subset in relation to the total number of CD34+ cells at the three conditions (pre-exercise, post-exercise, and apheresis) are displayed as median for each condition, with each axis scaled according to the highest third quartile value observed across all measurements per condition. The right half of the plot represents the viable cell subsets, while the left half displays the apoptotic cell subsets

The majority of CD34+ cells were viable, with viability rates of 93.0 (10.0) %, 95.2 (7.4) %, and 98.0 (3.1)% for pre-, post-exercise, and apheresis conditions, respectively, being comparable between conditions (all *p* > 0.05, Fig. 4). In all conditions expressed as relative to the total CD34+ cell number, most viable CD34+ cells also expressed CD45dim, with expressions ranging from 83.6 (11.0)% pre- to 84.7 (8.5)% post-exercise (*p* = 0.611) and increasing to 96.6 (2.8)% at apheresis (*p* = 0.002 to post-exercise). 45.3 (10.6)% of total CD34+ cells expressed the combination of CD38+CD133+ pre-exercise, which did not significantly change post-exercise (41.7 (10.5)%, *p* = 0.150), but also increased at apheresis to 75.6 (5.7)% of total CD34+ cells (*p* = 0.005 to post-exercise). The percentage of viable CD34+CD110+ cells (1.4 (1.2)% significantly decreased post-exercise (0.7 (0.8)%, *p* = 0.008) and was then comparable to the apheresis condition (0.3 (0.1)%, *p* = 0.097).

Apoptosis of total CD34+ cells and the CD34+CD45dim subset did not change with the acute exercise (both *p* > 0.05). However, apoptotic CD34+CD110+ (*p* = 0.015), and CD34+CD38+CD133+ (*p* = 0.013) expressions were significantly reduced post-exercise compared to baseline.

Viability and apoptosis of all other cell subsets were comparable between conditions expressed as percentage of total CD34+ cells (all *p* > 0.05, Table 1).

**Table 1:** Proportions of viable and apoptotic CD34+ cells as percent of mononuclear cells and respective cell subsets in percent of total CD34+ cells

Cell type (%)	Pre-exercise		Post-exercise		md [95%CI] (post-pre)		Apheresis		md [95%CI] (apheresis-post)	
	Viable	Apoptotic	Viable	Apoptotic	Viable	Apoptotic	Viable	Apoptotic	Viable	Apoptotic
CD34+	93.0 (10.0)	6.5 (9.6)	95.2 (7.4)	4.3 (7.3)	2.3 [-0.9, 5.4]	-2.2 [-5.2, 0.8]	98.0 (3.1)	1.7 (2.6)	2.8 [-2.0, 7.5]	-2.7 [-7.1, 1.7]
CD34 + CD45dim	83.6 (11.0)	4.8 (7.4)	84.7 (8.5)	1.8 (3.3)	1.2 [-3.9, 6.3]	-2.9 [-7.1, 1.2]	96.6 (2.8)**	1.4 (2.2)	11.8 [5.8, 17.8]	-0.4 [-2.8, 1.9]
CD34 + CD110+	1.4 (1.2)	1.4 (2.0)	0.7 (0.8)^§^	0.4 (0.7) ^§^	-0.6 [-1.1, -0. 2]	-1.0 [-2.0, 0. 1]	0.3 (0.1)	0.2 (0.2)	-0.5 [-1.1, 0.1]	-0.3 [-0.6, 0.1]
CD34 + CD38+CD133+	45.3 (10.6)	1.6 (2.6)	41.7 (10.5)	0.8 (1.2) ^§^	-3.6 [-8.7, 1.6]	-0.9 [-2.0, 0.3]	75.6 (5.7)**	0.9 (1.5)	33.9 [29.0, 38.7]	0.1 [-0.7, 1.0]
CD34 + CD38-	2.3 (1.4)	0.23 (0.3)	1.8 (0.8)	0.26 (0.4)	-0.5 [-1.6, 0.7]	0.02 [-0.3, 0.3]	1.2 (1.6)	0.02 (0.03)	-0.6 [-1.8, 0.6]	-0.24 [-0.5, 0.03]
CD34 + CD38-CD133+	0.1 (0.2)	0.1 (0.2)	0.2 (0.5)	0.2 (0.3)	0.1 [-0.2, 0.5]	0.1 [-0.2, 0.4]	0.9 (1.2)	0.01 (0.02)	0.6 [-0.4, 1.6]	-0.2 [-0.4, 0.07]

Data are presented as mean (standard deviation) for all three conditions; md [95%CI], mean difference [95%Confidence Interval]; ^§^*p* < 0.05 compared to pre-exercise, **p < 0.01 compared to post-exercise; ***p < 0.001 compared to post-exercise

### Viable and apoptotic CD34+ and subsets as concentration estimates

There were significant increases for viable CD34+ cell concentration estimates from pre- to post-exercise conditions (*p* = 0.003) as well as for their subsets CD34+CD45dim (*p* = 0.006), and CD34+CD38+CD133+ (*p* = 0.013), Table 2. The other cell subset concentration estimates were comparable between these conditions (all *p* > 0.05).

Compared to the post-exercise condition, viable CD34+ cells as well as most cell subsets showed significant increases in cell counts at apheresis (CD34+CD45dim, CD34+CD38+CD133+, both *p* < 0.001; CD34+CD110+, *p* = 0.004; CD34+CD38-, *p* = 0.005; CD34+CD38-CD133+, *p* = 0.041), Table 2.   e 

**Table 2:** Estimated concentrations of viable and apoptotic CD34+ cells and respective subsets in cells/µl

Cell type (cells/µl)	Pre-exercise		Post-exercise		md [95%CI] (post-pre)		Apheresis		md [95%CI] (apheresis-post)	
	Viable	Apoptotic	Viable	Apoptotic	Viable	Apoptotic	Viable	Apoptotic	Viable	Apoptotic
CD34+	1.951 (1.316)	0.089 (0.117)	3.333 (2.074) ^§§^	0.109 (0.189)	1.382 [0.617, 2.147]	0.020 [-0.049, 0.088]	63.293 (29.000) ***	1.128 (1.988)**	59.959 [40.317, 79.601]	1.019 [-0.342, 2.381]
CD34 + CD45dim	1.777 (1.235)	0.064 (0.092)	3.021 (2.008) ^§§^	0.043 (0.076)	1.244 [0.462, 2.025]	-0.020 [-0.067, 0.027]	62.469 (28.774) ***	0.965 (1.754)**	59.448 [39.934, 78.963]	0.921 [-0.317, 2.160]
CD34 + CD110+	0.022 (0.014)	0.020 (0.025)	0.020 (0.022)	0.012 (0.019)	-0.002 [-0.011, 0.007]	-0.008 [-0.017, 0.000]	0.117 (0.071)**	0.079 (0.114)*	0.097 [0.041, 0.154]	0.066 [-0.003, 0.136]
CD34 + CD38+CD133+	0.946 (0.626)	0.024 (0.032)	1.443 (0.912) ^§^	0.020 (0.032)	0.497 [0.132, 0.862]	-0.003 [-0.010, 0.003]	49.228 (23.398) ***	0.623 (1.118)*	47.785 [31.525, 64.045]	0.603 [-0.183, 1.389]
CD34 + CD38-	0.046 (0.055)	0.003 (0.004)	0.057 (0.036)	0.006 (0.008)	0.011 [-0.016, 0.038]	0.003 [-0.003, 0.009]	0.662 (0.802)**	0.015 (0.036)	0.605 [0.048, 1.162]	0.008 [-0.017, 0.034]
CD34 + CD38-CD133+	0.002 (0.003)	0.002 (0.003)	0.006 (0.010)	0.005 (0.007)	0.003 [-0.004, 0.011]	0.003 [-0.003, 0.009]	0.488 (0.642)*	0.003 (0.007)	0.483 [0.023, 0.942]	-0.002 [-0.010, 0.007]

Data are presented as mean (standard deviation) for all three conditions; md [95%CI], mean difference [95%Confidence Interval]; ^§^ *p* < 0.05 compared to pre-exercise, ^§§^ *p* < 0.01 compared to pre-exercise, * *p* < 0.05 compared to post-exercise, ** *p* < 0.01 compared to post-exercise; *** *p* < 0.001 compared to post-exercise

The acute exercise did not change any apoptotic cell concentration estimate, but apoptotic CD34+ and CD34+CD45dim cell number significantly increased from post-exercise to apheresis (*p* = 0.005, and *p* = 0.008, respectively). Apheresis also showed increased apoptotic CD34+CD38+CD133+ (*p* = 0.017) and CD34+CD110+ (*p* = 0.012) cell concentration estimates compared to post-exercise.

### Correlations of donor CD34+ cell concentration estimates to donor body composition, inflammation, CRF and physical activity

**Fig. 5:**
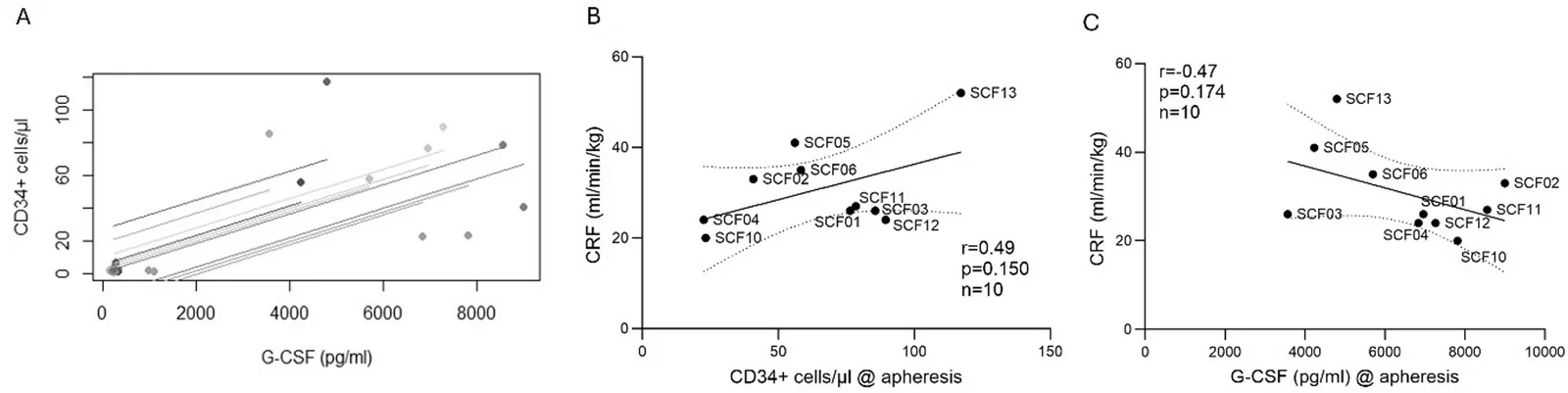
Correlation between donor variables. Donor total CD34+ cell concentration estimate was positively related to donor G-CSF at pre-, post-exercise and apheresis (r_rm_= 0.84, *p* < 0.001, df = 19) (**A**). Donor CRF was not associated with CD34+ cell number at apheresis (*r* = 0.49, *p* = 0.150, *n* = 10) (**B**), or G-CSF at apheresis (*r* = -0.47, *p* = 0.174, *n* = 10, **C**). *G-CSF* granulocyte colony-stimulating factor; *CRF* cardiorespiratory fitness; *SCF*,StemCellFit donor study ID;

Donor BMI showed no significant relationship with (apoptotic) CD34+ cell concentration estimates at apheresis, but donor WHR was negatively associated with live CD34+, live CD34+CD45dim, and live CD38+CD133+ cells/µl (*r *= -0.677, *p* = 0.032; *r *= -0.677, *p* = 0.031; *r *= -0.643, *p* = 0.045; respectively, all *n* = 10). Donor CD34+ cell concentration estimates and G-CSF showed a very strong association over all conditions (r_rm_=0.84, *p* < 0.001, df = 19, Fig. 5A). Donor CRF was neither significantly related to donor CD34+ cells/µl at the day of apheresis (*r* = 0.49, *p* = 0.150, respectively, *n* = 10, Fig. 5B), nor to G-CSF at the day of apheresis (*r *= -0.47, *p* = 0.174, respectively, *n* = 10, Fig. 5C). Biomarkers of physical activity (total MET, rho = 0.06, *p* = 0.433, *n* = 10; avacc, *r* = 0.21, *p* = 0.612, *n* = 8; ig, *r* = 0.30, *p* = 0.474; *n* = 8) were not significantly associated with (apoptotic) CD34+ cell concentration estimates at apheresis.

### Correlations of donor CRF and inflammation to recipient engrafting cells and quality of life at day-30 post-transplant

**Fig. 6:**
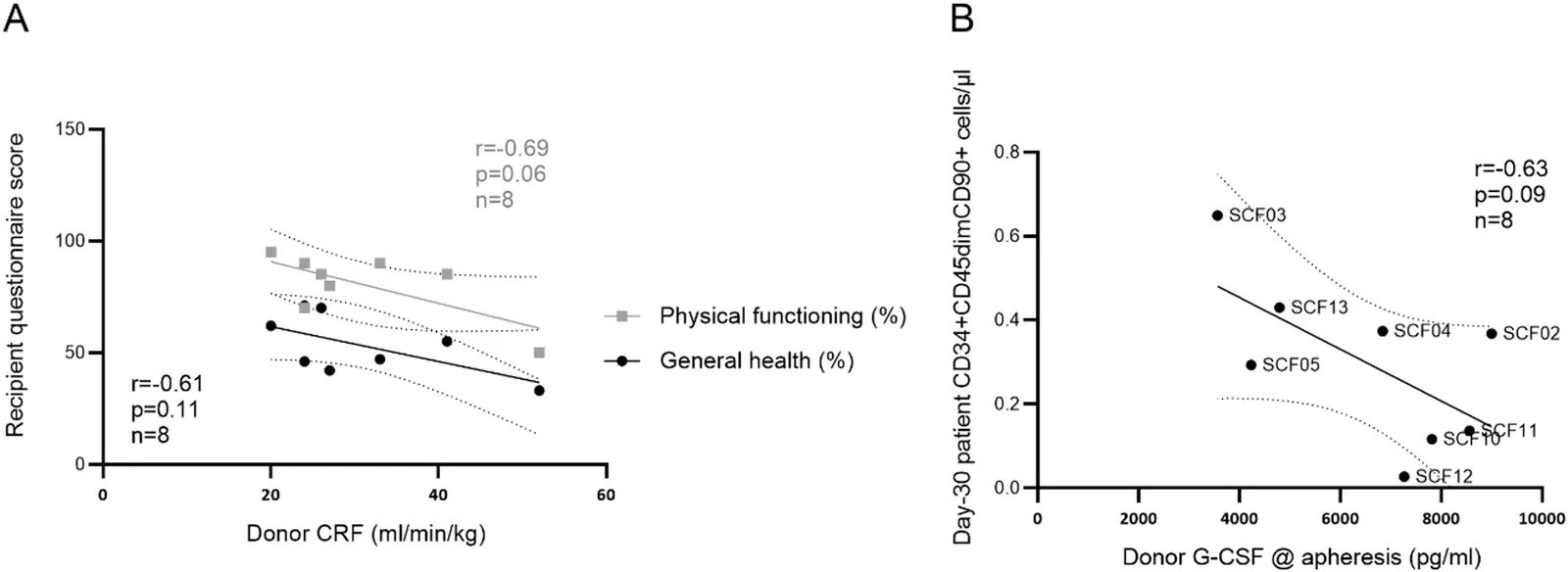
Correlations between donor and recipient variables. Donor CRF **A** showed a negative association with recipient physical functioning (*r* = -0.69, *p* = 0.070, *n* = 8) and general health (*r *= -0.61, *p* = 0.106, *n* = 8). Donor G-CSF at apheresis **B** showed an inverse relationship to recipient engrafting CD34+45dimCD90+ cell number at day ~ 30 post-transplant (*r *= -0.63, *p* = 0.095, *n* = 8). Results were non-significant; *G-CSF*, granulocyte colony-stimulating factor; *CRF*, cardiorespiratory fitness

Donor CRF showed a moderate negative relation to recipient QoL outcome parameters such as physical functioning (*r* = -0.69, *p* = 0.070, *n* = 8, Fig. 6A) and general health (*r* = -0.61, *p* = 0.106, *n* = 8, Fig. 6A) at day ~ 30 post-transplant, although associations were non-significant. At the same time, donor G-CSF at the day of apheresis revealed a moderate, although non-significant, inverse relationship with recipient engrafting CD34+CD45dimCD90+ cells/µl (*r *= -0.63, *p* = 0.095, *n* = 8, Fig. 6B).

### Correlations of donor CD34+ cell concentration estimates to recipient engrafting cells at day ~ 30 post-transplant

**Fig. 7:**
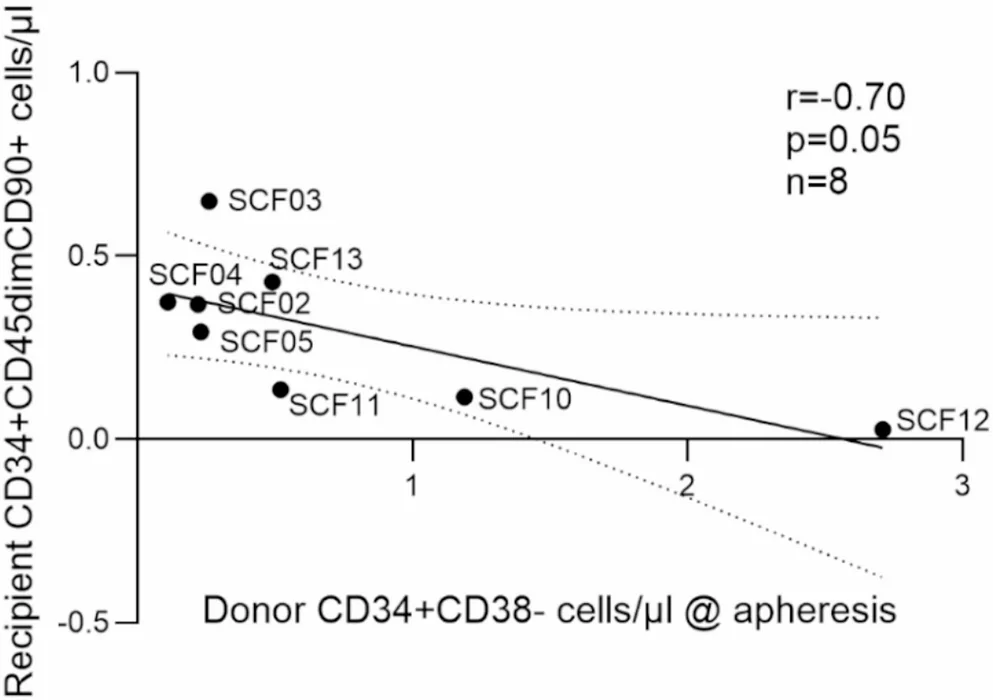
Correlation between a primitive donor cell subgroup at HSC collection and recipient engrafting cells at day ~ 30 post-transplant. Donor CD34+CD38- cell number was significantly negatively associated with recipient engrafting CD34+CD45dimCD90+ cells/µl (*r *= -0.70, *p* = 0.05, *n* = 8)

Donor CD34+CD38- cell concentration estimates at the day of apheresis were significantly negatively related to recipient engrafting CD34+CD45dimCD90+ cells/µl at day ~ 30 post-transplant (*r *= -0.70, *p* = 0.05, *n* = 8, Fig. 7).

## Discussion

This study is the first to compare the mobilization of mature immune cells, CD34+ HSC and their subgroups as well as the extent of cell apoptosis and viability in response to G-CSF and acute exercise. Both interventions increased total CD34+ cell levels in peripheral blood, but with distinct cellular patterns. When compared to the other mobilization method, G-CSF primarily mobilized higher proportions of viable CD34⁺CD45dim and CD34 + CD38+CD133 + cells, while exercise induced equal mobilization of platelet precursors (CD34+CD110+) and apoptotic primitive progenitors (CD34 + CD38- and CD34 + CD38-CD133+). Apoptosis rates among CD34+ cells and their subsets were similar between the two methods. Only G-CSF induced excessive mobilization of IG. Although there was no hint that donor CRF would influence inflammation or CD34+ mobilization on the day of apheresis, it showed moderate, although non-significant, associations with recipient physical functioning and generalhealth.

In this study, donors exhibited on average a comparable white blood cell count at apheresis (49*10^3^/µl) to that reported in the literature (Allen and Conry-Cantilena 2019). On the contrary, CD34+ cell concentrations increased on average to 64.8 cells/µl at apheresis, which is lower than the previously described range of 84 to 119 CD34+ cells/µl (Allen and Conry-Cantilena 2019). This may be attributable to heterogeneity within the donor population, variations in mobilization protocols and G-CSF dosing or differences in the definition of true CD34+ cells (Sutherland et al. 1996), which in this study was based on the presence of the CD34+ surface marker combined with a low side scatter (< 300 units). The observed 1.6 to 1.8-fold increase in total CD34+ cells and most subsets from pre- to post-exercise is consistent with previous research (Landers-Ramos et al. 2019; Schmid et al. 2021; Niemiro et al. 2017). All measured CD34+ cell subsets showed a substantial increase in concentration at apheresis compared to the post-exercise condition.

HSC collection composition is crucial to the success of engraftment, with primitive HSC subsets such as CD34+CD38- and the amount of IG playing a particularly important role (Henon et al. 2001; Tecchio and Cassatella 2021). While the post-exercise cell surface marker expression profile of viable CD34+CD38- cells and the even more primitive viable CD34+CD38-CD133 + subset roughly resembles the one from apheresis, there are notable increases in relative proportions of viable CD34+CD45dim and CD34+CD38+CD133 + cells at apheresis compared to post-exercise. This may be due to different mobilization mechanisms (Salhotra et al. 2023; Agha et al. 2018). Mobilization intensity and duration differ significantly between G-CSF and acute exercise (Simpson et al. 2017; Gustafson et al. 2021). G-CSF induces a high-intensity and sustained mobilization of HSC and IG by disrupting bone marrow retention signals (Theyab et al. 2021; Endo-Matsubara et al. 2004), resulting in elevated cell levels in circulation for hours to days. In contrast, post-acute exercise triggers lower-intensity, transient mobilization driven by physiological stressors such as catecholamine release and increased blood flow (Agha et al. 2018). This response is short-lived, with HSC levels and IG typically peaking within minutes to a few hours after exercise (Kruger et al. 2015). During apheresis, more primitive HSC, but also excessive IG are collected due to strong pharmacological mobilization (Pederzoli et al. 2024) while post-exercise mobilization additionally triggers endothelial progenitor cells and less primitive HSC subsets (Schmid et al. 2021; Medina et al. 2017). However, it is important to note that the expression levels of viable CD34+CD38-, CD34+CD38-CD133+, and CD34+CD110+ cells are not inferior following exercise compared to apheresis and that IG - a response to inflammation - are only slightly increased by the acute exercise. This is a critical consideration when evaluating acute exercise as adjuvant treatment for HSC collection in terms of donor inflammation (Bogeska et al. 2022), overall patient survival (Henon et al. 2001), long-term multilineage engraftment, and platelet reconstitution (Baker et al. 2017; Sartor et al. 2007; Camacho Villa et al. 2012).

Interestingly, donor body composition also appeared to influence HSC collection characteristics: a lower WHR - which is often associated with higher CRF (Lipowicz et al. 2025) - was related to higher concentrations of viable CD34+, CD34+CD45dim, and CD38+CD133+ cells at apheresis.

Apoptosis of HSC in this context has not been studied in detail. Interestingly, the results of this study suggest that apoptotic CD34+ cells and their subset proportions do not significantly differ between post-exercise and apheresis conditions. Acute exercise as physiological stressor and G-CSF as pharmacological intervention appear to equally impact regulated cell death in the HSC collection, which is a good sign regarding cell fitness and viability when considering exercise as adjuvant therapy for HSCT (Afreen et al. 2018; Emmons et al. 2016). Combining exercise with G-CSF as mobilizing agent to enhance the yield of key CD34+ subsets is unlikely to compromise cell quality (Wadley et al. 2025).

However, the difference in mobilized cell concentrations between physical exercise and G-CSF is considerable, with total CD34+ cell concentrations and most of their subpopulations increasing approximately 2-fold following exercise and up to 50-fold during apheresis. Interestingly, the apoptotic CD34+CD38-CD133+ subset important for long-term hematopoietic reconstitution in the recipient responds with a similar mobilization magnitude to both endogenous (exercise-induced) and exogenous (G-CSF-induced) regimens, although overall mobilization efficacy between these regimens highly differs. This may partly explain why the kinetics of endogenous mobilization via the non-invasive exercise approach can be viewed as more favorable, especially since cell viability is not negatively affected. Moreover, a single bout of physical exercise, compared to G-CSF-induced mobilization, beneficially primes the mature immune system - inducing fewer regulatory T cells and a greater number of cytotoxic lymphocytes - which could enhance the efficacy of adoptive cell transfer following HSCT (Heinemann et al. 2020). It is speculated that this is especially valuable in individuals with lower physical fitness and/or activity levels, since moderately fit individuals showed clearer improvements in T-cell mobilization and function (Heinemann et al. 2020). Notably, regular long-term exercise is known to counter T-cell immune senescence (Turner and Brum 2017). Our data add to this knowledge by suggesting that donor CRF, a modifiable factor associated with immune function and mortality, is related to recipient health status. Interestingly, CRF has recently been hypothesized as a useful predictor of HSC mobilization and outcomes after HCT (McDougal et al. 2025). Our findings partially support this hypothesis, suggesting that lower donor CRF - though likely unrelated to inflammation levels on the day of apheresis respective CD34+ cell concentration - is probably associated with better recipient general health and physical functioning at day ~ 30 post-transplant. This raises the hypothesis that less fit donors may, counterintuitively, be more beneficial than fitter ones. It could be speculated that a less reactive allograft may reduce the risk of graft-versus-host disease (Zaghi et al. 2019). Or that a slower immune recovery might allow the recipient’s body to adapt more gradually to the graft (van den Brink et al. 2015), albeit also the opposite is discussed (McDougal et al. 2025).

Interestingly, our results also bring up the question, whether an excess of donor G-CSF or early CD34+CD38- at HSC collection could potentially impair the engraftment of the most potent long-term HSC, leading to e.g. fewer sustained CD34+CD45dimCD90+ cells in the recipient. Along the same line, recent research suggests that donor stem cell graft dose and cellular composition are potential determinants of donor-derived clonal expansion after allogeneic HSCT (Stiehl 2024), and that post-transplant G-CSF hinders engraftment of human HSC by intensifying DNA damage (Araki et al. 2025).

### Strengths and limitations

Our study design has several notable strengths. By directly comparing both exogenous and endogenous HSC mobilization strategies within the same donor, we minimize data variability. Moreover, the inclusion of detailed HSC analyses - such as the assessment of regulated cell death - addresses an important gap in stem cell mobilization research. Further, the acute exercise was used as a means to mobilize HSC, but also to assess CRF, limiting donor burden and additional testing. CRF assessment was also performed in close temporal proximity to the apheresis procedure.

However, our study does have limitations, the most significant being the small sample size, despite a recruitment period of 1.5 years. Recruitment at a single center with related donors was difficult due to logistical, language, and psychological barriers. Challenges included psychological stress due to having a relative with a life-threatening illness, stress related to the potential risks of donation, possible inability to work, and/or limited willingness to undergo additional testing. In addition, the Swiss ethics committee imposes strict regulations concerning donor burden and prohibits any additional responsibilities beyond those associated with HSC donation. As a result, donors could not be approached personally or outside of the scheduled donor evaluation at the Blood Transfusion Center. Furthermore, in some cases, the intended recipients passed away between the donor evaluation and the HSC collection, further limiting enrollment. One recipient died following HSC transplantation, and another was underage, making any follow-up investigations impossible. In two cases, donors were unable to wear the accelerometer for the required duration: one donor needed to care for the respective recipient (a child), and for the other, the wear time would have overlapped with the initiation of G-CSF treatment. Lacking any donor follow-up analysis, this study may not fully reveal mechanistic links between donor CRF, inflammation, physical activity and HSC mobilization or recipient outcome. Lastly, although dual-platform flow cytometry approaches may introduce greater variability in absolute cell quantification compared with single-platform methods, we deliberately chose this approach to align with our laboratory workflow, which was carefully overseen. By relying on independently quality-controlled differential leukocyte counts from a calibrated hematology analyzer, we ensured consistency with routine laboratory standards while maintaining standardized immunophenotypic analysis by flow cytometry. All measurements were conducted under strict internal and external quality control procedures to minimize potential variability.

## Conclusions and future research

Although exogenous and endogenous mobilization strategies differ in both HSC collection composition and mobilization magnitude, acute exercise may serve as a favorable adjunct to support G-CSF-induced HSC collection and recipient engraftment, without exerting a negative impact on regulated cell death. Future research should investigate the feasibility and effectiveness of combining these two approaches to optimize mobilization efficiency, enhance HSC collection quality, and improve engraftment success. In addition, studies should assess whether CRF constitutes an additional determinant of donor suitability and contributes to better recipient outcomes. However, numerous operational aspects must first be clarified, with careful consideration of donor safety.

## Supplementary Information

Below is the link to the electronic supplementary material.

Supplementary Material 1

## Data Availability

The datasets generated during the current study are available from the corresponding author on reasonable request.
