# Supplementary material for: Cellular profile of hematopoietic stem cells mobilized by exercise versus G-CSF in healthy related donors
Source: Eur J Appl Physiol. 2026 Apr 10;126(7):4113–26. doi: 10.1007/s00421-026-06224-7 (PMC13661763; doi:10.1007/s00421-026-06224-7)
Supplement: Supplementary file 1 [file 421_2026_6224_MOESM1_ESM.docx]

**SUPPLEMENT to**

**Cellular Profile of Hematopoietic Stem Cells Mobilized by Exercise versus G-CSF in Healthy Related Donors**

Elias Siebold^a^ (0009-0000-1186-6019), Laura Infanti^b,c^ (0000-0001-7241-426), Gregor T. Stehle^b,c^, Debbie Maurer^a^, Egbert T. Ritter^a^, Michael Medinger^c,d^ (0000-0003-0427-7747), Francesca Matteazzi^c^, Arno Schmidt-Trucksäss^a^ (0000-0002-4662-3911), Andreas Holbro^b,c§^ (0000-0002-2394-2223), and Julia M. Kröpfl^*a§^ (0000-0002-9613-7268)

^a^Division of Sport and Exercise Medicine, Department of Sport, Exercise and Health, University of Basel, Grosse Allee 6, 4052 Basel, Switzerland

^b^Blood Transfusion Center North-Western Switzerland, Swiss Red Cross, Hebelstrasse 10, 4056 Basel, Switzerland

^c^Clinic of Hematology, University Hospital Basel, Petersgraben 4, 4031 Basel, Switzerland

^d^Clinic of Internal Medicine III: Oncology, Hematology and Palliative Medicine, Diakoniestrasse 10, Schwäbisch Hall, Germany

^§^ these authors contributed equally to the manuscript.

*** Correspondence**
Julia Maria Kröpfl, PhD

[juliamaria.kroepfl@unibas.ch](mailto:juliamaria.kroepfl@unibas.ch)

| **ID** | **Donor details (sex, age, BMI)** | **Recipient details (sex, age, BMI)** | **Indication for allo-HSCT** |
| --- | --- | --- | --- |
| SCF01 | F, 39, 21.2 | underage | n.a. |
| SCF02 | F, 25, 22.5 | M, 23, 22.8 | Acute myeloid leukemia |
| SCF03 | M, 61, 31.9 | M, 65, 27.1 | Primary myelofibrosis |
| SCF04 | F, 45, 30.7 | M, 43, 28.0 | Myeloproliferative syndrome |
| SCF05 | M, 51, 23.9 | M, 47, 25.1 | Acute myeloid leukemia |
| SCF06 | M, 31, 27.2 | death | n.a. |
| SCF10 | M, 75, 29.6 | F, 73, 19.9 | Myelodysplastic syndrome |
| SCF11 | F, 20, 26.2 | F, 22, 23.6 | Acute myeloid leukemia |
| SCF12 | M, 43, 25.5 | M, 47, 23.8 | Acute myeloid leukemia |
| SCF13 | M, 19, 20.9 | F, 60, 33.0 | Acute myeloid leukemia |

*Table S1: Donor and recipient characteristics; allo-HSCT, allogeneic hematopoietic stem cell transplantation; M, male; F, female; n.a., not available*
